# A very early diagnosis of Alstrӧm syndrome by next generation sequencing

**DOI:** 10.1186/s12881-020-01110-1

**Published:** 2020-09-01

**Authors:** Leonardo Gatticchi, Jan Miertus, Paolo Enrico Maltese, Simone Bressan, Luca De Antoni, Ludmila Podracká, Lucia Piteková, Vanda Rísová, Mari Mällo, Kaie Jaakson, Kairit Joost, Leonardo Colombo, Matteo Bertelli

**Affiliations:** 1 Department of Experimental Medicine, Laboratory of Biochemistry, University of Perugia, Perugia, Italy; 2 Génius n. o, Trnava, Slovakia; 3 MAGI’s Lab, Genetic Testing Laboratory, Via Delle Maioliche 57/D, 38068 Rovereto, TN Italy; 4 MAGI Euregio, Via Maso della Pieve, 60/A, 39100 Bolzano, Italy; 5 Department of Pediatrics, National Institute for Sick Children, Commenius University, Bratislava, Slovakia; 6 Institute of Histology and Embryology, Faculty of Medicine, Commenius University, Bratislava, Slovakia; 7 Asper Biogene LLC, Tartu, Estonia; 8 Department of Ophthalmology, San Paolo Hospital, University of Milan, Milan, Italy

**Keywords:** Alström syndrome, *ALMS1*, Next generation sequencing, Case report

## Abstract

**Background:**

Alström syndrome is a rare recessively inherited disorder caused by variants in the *ALMS1* gene. It is characterized by multiple organ dysfunction, including cone-rod retinal dystrophy, dilated cardiomyopathy, hearing loss, obesity, insulin resistance, hyperinsulinemia, type 2 diabetes mellitus and systemic fibrosis. Heterogeneity and age-dependent development of clinical manifestations make it difficult to obtain a clear diagnosis, especially in pediatric patients.

**Case presentation:**

Here we report the case of a girl with Alström syndrome. Genetic examination was proposed at age 22 months when suspected macular degeneration was the only major finding. Next generation sequencing of a panel of genes linked to eye-related pathologies revealed two compound heterozygous variants in the *ALMS1* gene. Frameshift variants c.1196_1202del, p.(Thr399Lysfs*11), rs761292021 and c.11310_11313del, (p.Glu3771Trpfs*18), rs747272625 were detected in exons 5 and 16, respectively. Both variants cause frameshifts and generation of a premature stop-codon that probably leads to mRNA nonsense-mediated decay. Validation and segregation of *ALMS1* variants were confirmed by Sanger sequencing.

**Conclusions:**

Genetic testing makes it possible, even in childhood, to increase the number of correct diagnoses of patients who have ambiguous phenotypes caused by rare genetic variants. The development of high-throughput sequencing technologies offers an exceptionally valuable screening tool for clear genetic diagnoses and ensures early multidisciplinary management and treatment of the emerging symptoms.

## Background

Alström syndrome (AS; OMIM #203800) is a rare recessively inherited disorder caused by loss-of-function variants in the *ALMS1* gene [1]. It is typically associated with early onset and rapid development of cone-rod retinal dystrophy, usually with nystagmus and strong photophobia. Retinal degeneration may also be accompanied by dilated cardiomyopathy, congestive heart failure and bilateral sensorineural hearing impairment [2, 3]. Other clinical manifestations progress slowly, leading to multiple organ dysfunction due to the concurrence of metabolic complications like obesity, insulin resistance, hyperinsulinemia, dyslipidemia and type 2 diabetes mellitus, as well as widespread fibrosis that can lead to kidney, respiratory, pancreatic and liver failure [4–6].

*ALMS1* is a 23-exon gene located on chromosome 2p13; it encodes a ubiquitously expressed protein product associated with the centrosome and basal body of primary cilia [7, 8]. Recent studies on the functions of ALMS1 suggest that AS is in the class of human genetic disorders linked to ciliary dysfunction, known as ciliopathies [9–11]. In fact, ALMS1 seems to be required for correct formation and function of primary cilia. Cilia are hair-like cell projections that occur in many types of cell and are involved in the sensory processes of terminally differentiated cells, through special signaling transduction pathways [12–14]. The exact function of ALMS1 is being studied but roles in cytoskeleton and microtubule organization, endosomal and ciliary transport, cell cycle regulation of neonatal cardiomyocytes, and blood pressure and renal homeostasis were recently proposed [4, 15–21]. However, isoforms of ALMS1 with non-centrosomal sites of localization have been described, suggesting extra-ciliary functions as well [22–25].

The pleiotropic array of functions displayed by ALMS1 in a variety of different cell types may partly explain the heterogeneous clinical manifestations of AS, which in the end make it difficult to obtain a clear diagnosis, especially in children. The current diagnostic criteria for AS require a combination of major and minor features, according to the age of onset [3]. Indeed, the phenotype of AS partly overlaps with that of the other main ciliopathy, Bardet-Biedl syndrome (BBS), and differential diagnosis is often made by excluding clinical manifestations specific to BBS [26]. Atypical cases with non-syndromic retinal dystrophy or isolated cardiomyopathy due to variants in *ALMS1* have also been reported [27, 28]. The development of high-throughput sequencing technologies, such as whole-genome or exome sequencing, offers an exceptionally valuable screening tool for obtaining clear genetic diagnoses and identifying disease-causing gene variants. To date, about 1000 cases of AS have been confirmed worldwide, making a prevalence of 1–9 per million individuals (http://www.orpha.net). More than 200 variants in *ALMS1* have been described, mainly involving frameshift or nonsense variants that introduce premature stop codons [29]. Most of the *ALMS1* variants detected are clustered in exons 8 (49% of all known variants), 10 and 16 [29–33], although other variants have been reported in exon 5 [34–36] and others in intronic regions [29, 33, 37, 38].

Here we report the case of a 22-month-old girl with Alström syndrome. Early genetic diagnosis was achieved by an optimized next generation sequencing approach, targeting a panel of genes linked to eye-related pathologies that revealed two compound heterozygous variants in the *ALMS1* gene. The importance of preemptive genetic screening and future prospects for treating Alström syndrome are also discussed.

## Case presentation

The proband was born at term (week 39) by caesarean section due to fetal position, without complications (Apgar score at 5′ = 10). Weight (2720 g), length (47 cm) and head circumference (33 cm) were normal. Subsequent growth and development were normal (e.g. w = 5970 g, l = 61 cm, hc = 40.5 cm at 4 months). A small jugular hemangioma was noticed at age 6 months by the pediatrician. The infant recovered from several episodes of acute bronchitis that have continued to exacerbate. At age 1 year an ophthalmologist was consulted because her parents noticed photophobia (squinting) and random hitting of objects. The specialist suspected macular degeneration and found convergent concomitant strabismus of the right eye. Fluorescein angiography showed a patchy hyperfluorescent fundus, adequate filling of the vascular bed, macula with thinner or centrally patchy retinal pigmented epithelium with surrounding spots/patches of window-type defect and initial “bull’s eye” features, in line with a diagnosis of maculopathy or cone dystrophy.

At age one year, the infant contracted acute *Haemophilus influenzae*-positive pneumonia. At age 22 months genetic examination was proposed.

Genomic DNA was extracted from 200 μl whole blood using a QIAamp DNA Mini Kit (Qiagen GmbH, Hilden, Germany). Library preparation and sample enrichment were performed with an Illumina TruSight One kit. Next generation sequencing (NGS) was performed on an Illumina NextSeq 500 system (Illumina, San Diego, CA, USA). Variants differing from hg19 reference sequence were called with BWA2.1 in Illumina BaseSpace. Data annotation for 277 genes associated with hereditary eye diseases was performed with programs Varvis (Limbus Medical Technologies GmbH, Germany) and Alamut Visual (Interactive Biosoftware, France) and rare variants (frequency less than 1%) were filtered. Pathogenic variants of human *ALMS1* (RefSeq: NM_015120.4) were confirmed with PCR and Sanger sequencing on a 3730xl DNA analyser (Applied Biosystems, Foster City, CA, USA). Sequences were analysed with BioEdit (Ibis Therapeutics, CA, USA) and ChromasPro (Technelysium Pty Ltd., Australia). Genetic variants were submitted to the Human Gene Mutation Database (HGMD), Leiden Open Variation Database (LOVD), 1000 Genome Project and the Exome Aggregation Consortium (ExAC) database. The pathogenicity of each variant was checked using the on-line software VarSome (https://varsome.com/) [39]. Because of the non-specificity of the clinical picture and diagnosis, all 277 “eye exome” genes were tested: *ABCA4*, *ABCB6*, *ABCC6*, *ABHD12*, *ACBD5*, *ADAM9*, *ADAMTS18*, *ADGRV1*, *AGK*, *AHI1*, *AIPL1*, *ALMS1* (excluding exon 8), *ARL13B*, *ARL6*, *B3GLCT*, *BBS1*, *BBS10*, *BBS12*, *BBS2*, *BBS4*, *BBS5*, *BBS7*, *BBS9*, *BCOR*, *BEST1*, *BFSP2*, *BMP4*, *C19ORF12*, *C1QTNF5*, *C2orf71*, *C8ORF37*, *CA4*, *CABP4*, *CACNA1F*, *CACNA2D4*, *CC2D2A*, *CDH23*, *CDH3*, *CDHR1*, *CEP290*, *CEP41*, *CERKL*, *CFH*, *CHM*, *CHMP4B*, *CHST6*, *CIB2*, *CLN3*, *CLN5*, *CLN6*, *CLN8*, *CLRN1*, *CNGA1*, *CNGA3*, *CNGB1*, *CNGB3*, *CNNM4*, *COL11A1*, *COL11A2*, *COL2A1*, *COL4A1*, *COL8A2*, *COL9A1*, *COL9A2*, *CRB1*, *CRX*, *CRYAA*, *CRYAB*, *CRYBA1*, *CRYBA4*, *CRYBB1*, *CRYBB2*, *CRYBB3*, *CRYGB*, *CRYGC*, *CRYGD*, *CRYGS*, *CTDP1*, *CTSD*, *CYP1B1*, *CYP4V2*, *DCN*, *DHDDS*, *EFEMP1*, *ELOVL4*, *EPHA2*, *EYS*, *FAM161A*, *FLVCR1*, *FRAS1*, *FREM1*, *FREM2*, *FSCN2*, *FTL*, *FYCO1*, *FZD4*, *GALK1*, *GALT*, *GDF3*, *GDF6*, *GFER*, *GIPC3*, *GJA1*, *GJA3*, *GNAT1*, *GNAT2*, *GNPTG*, *GPR143*, *GPR179*, *GRIP1*, *GRK1*, *GRM6*, *GRN*, *GSN*, *GUCA1A*, *GUCA1B*, *GUCY2D*, *HARS*, *HCCS*, *HMX1*, *HSF4*, *IDH3B*, *IFT140*, *IMPDH1*, *IMPG2*, *INVS*, *ITM2B*, *IQCB1*, *JAG1*, *JAM3*, *KCNJ13*, *KCNV2*, *KIF11*, *KIF7*, *KLHL7*, *KRT12*, *KRT3*, *LAMA1*, *LCA5*, *LIM2*, *LRAT*, *LRP5*, *LZTFL1*, *MAK*, *MERTK*, *MFN2*, *MFRP*, *MFSD8*, *MIP*, *MKKS*, *MKS1*, *MTTP*, *MVK*, *MYO7A*, *MYOC*, *NAA10*, *NDP*, *NHS*, *NMNAT1*, *NPHP1*, *NPHP3*, *NPHP4*, *NR2E3*, *NRL*, *NYX*, *OAT*, *OFD1*, *OPA1*, *OPA3*, *OPN1MW*, *OTX2*, *PANK2*, *PAX2*, *PAX6*, *PCDH15*, *PDE6A*, *PDE6B*, *PDE6C*, *PDE6G*, *PDE6H*, *PDZD7*, *PEX7*, *PHYH*, *PIKFYVE*, *PITPNM3*, *PITX2*, *PITX3*, *PLA2G5*, *PPT1*, *PRCD*, *PRDM5*, *PROM1*, *PRPF3*, *PRPF31*, *PRPF6*, *PRPF8*, *PRPH2*, *PRSS56*, *RAB28*, *RAX2*, *RBP3*, *RBP4*, *RD3*, *RDH12*, *RDH5*, *RGR*, *RGS9*, *RGS9BP*, *RHO*, *RIMS1*, *RLBP1*, *ROM1*, *RP1*, *RP1L1*, *RP2*, *RP9*, *RPE65*, *RPGR* (excluding ORF15), *RPGRIP1*, *RPGRIP1L*, *RS1*, *SAG*, *SDCCAG8*, *SEMA4A*, *SIX6*, *SLC24A1*, *SLC45A2*, *SLC4A11*, *SMOC1*, *SNRNP200*, *SOX2*, *SPATA7*, *STRA6*, *TACSTD2*, *TCTN1*, *TCTN2*, *TDRD7*, *TEAD1*, *TGFBI*, *TIMM8A*, *TIMP3*, *TMEM126A*, *TMEM138*, *TMEM216*, *TMEM237*, *TMEM67*, *TOPORS*, *TPP1*, *TREX1*, *TRIM32*, *TRPM1*, *TSPAN12*, *TTC21B*, *TTC8*, *TULP1*, *TYR*, *TYRP1*, *UBIAD1*, *UNC119*, *USH1C*, *USH1G*, *USH2A*, *VAX1*, *VCAN*, *VIM*, *VPS13B*, *VSX1*, *VSX2*, *WDPCP*, *WDR19*, *WFS1*, *WHRN*, *YAP1*, *ZEB1*, *ZNF469*, *ZNF513*, *ZNF644*.

The proband’s parents agreed to this genetic test and signed written informed consent to use of the anonymized clinical and genetic results for research. All genetic and clinical data was collected as part of routine diagnosis and does not require ethical approval.

**Fig. 1:**
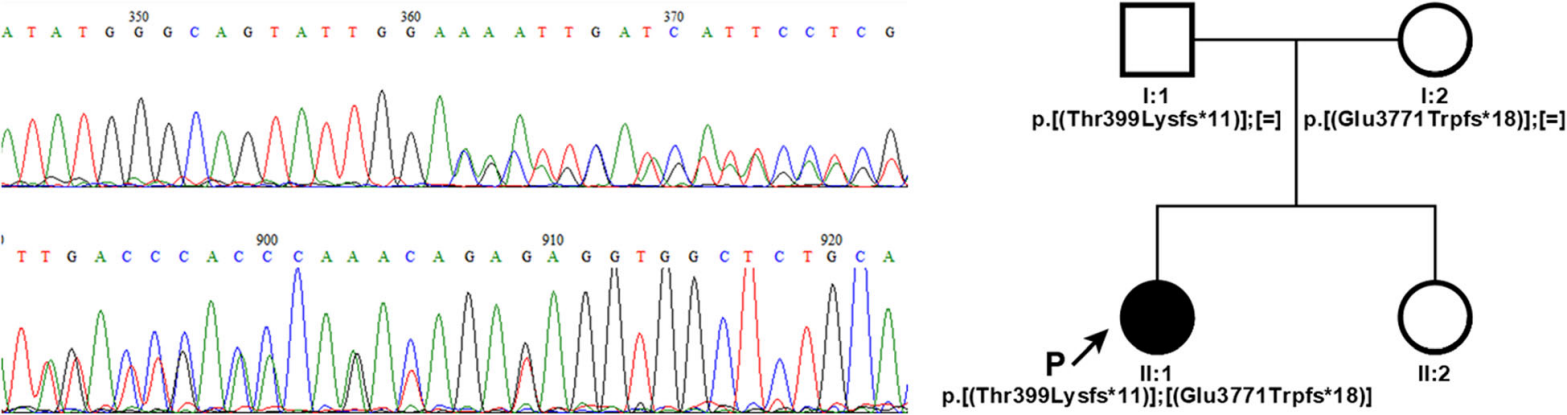
Sequence chromatograms of the variants identified in the proband. The chromatograms show the c.1196_1202del: p.(Thr399Lysfs*11) (upper panel) and the c.11310_11313del: p.(Glu3771Trpfs*18) (lower panel) heterozygous deletions. The family pedigree shows the compound heterozygous state of these two variants in the proband

Genetic testing revealed two compound heterozygous variants of the *ALMS1* gene in *trans* conformation: i) a paternally inherited deletion in exon 5 c.1196_1202del, p.(Thr399Lysfs*11), rs761292021; ii) a maternally inherited deletion in exon 16 c.11310_11313del, (p.Glu3771Trpfs*18), rs747272625 (Fig. 1). The first variant results in an amino acid substitution of residue 399 (Thr/Lys) and a frameshift leading to the creation of a premature stop codon after 11 residues. The second variant produces a similar result, characterized by a Glu/Trp substitution at position 3771 and generation of a premature stop codon after 18 residues. Validation and segregation of the *ALMS1* variants were confirmed by Sanger sequencing. A healthy 5-year-old sister of the proband tested negative to segregation analysis. DNA sequencing of the proband and the related summary of the 277 “eye exome” genes can be found as Additional files 1 and 2 respectively; Sanger sequencing data of the proband and their relatives can be found as Additional file 3.

On diagnosis of Alström syndrome, a very detailed report of the significance of this result was prepared (post-test genetic counselling) and a specific preventive diagnostic plan was formulated. The infant then underwent cardiological examination: ultrasonography showed borderline heart dimensions for weight, decreased function of left ventricle, no signs of true dilative cardiomyopathy or heart failure; no therapy but monitoring every 6–12 months was prescribed; picture unchanged after 1 year. Neurological examination was initially normal; since follow-up at age 3 years showed continued need of diapers, urological evaluation was proposed. Linguistic and graphic apraxia (intellectual disabilities) subsequently appeared with attention deficit and hyperkinetic behaviour; flat feet were recorded. Routine blood tests showed hypercholesterolemia (total Ct = 5.02 mmolL^− 1^) and hypertriglyceridemia (1.74 mmolL^− 1^) as defined by levels superior than 4.8 mmolL^− 1^ and 1.64 mmolL^− 1^, respectively; a low calorie, low fat, omega-3 rich diet was proposed. Ear, nose and throat evaluation showed chronic catarrhal inflammation of the middle ear (“glue ear”: the mother retrospectively reported having to raise her voice to speak to her daughter) treated with surgical fitting of grommets. Lung evaluation showed bronchial asthma to be treated with beta-mimetics and antibiotics if needed; electron microscope histopathological examination of a laryngobronchoscopy biopsy specimen showed a decreased number of cilia, many substituted by microvilli (basal corpuscle present), normal microtubule conformation and conserved dynein forks. The ophthalmologist prescribed dark orange glasses with correction for hypermetropia (+4D/+3D) and astigmatism. Perigenital mycosis developed at a later age.

## Discussion and conclusions

Alström syndrome (AS) is a rare multisystemic disorder caused by mutations in the *ALMS1* gene. Clinical manifestations include onset of cone-rod retinal degeneration in the first year of life. The retinal dystrophy usually progresses to bilateral blindness and may be accompanied by additional phenotypes like dilated cardiomyopathy, hearing loss, obesity, severe insulin resistance and type 2 diabetes mellitus. Although the incidence of these complications is high, AS patients develop the various symptoms in an age-dependent manner, in some cases in early infancy but usually in childhood or adolescence. Retinal dystrophy is usually the first sign that draws attention to AS, although non-syndromic retinal dystrophy has been reported in association with *ALMS1* variants [27], or in association with hearing loss mimicking Usher syndrome, the most prevalent form of syndromic retinitis pigmentosa to consider for differential diagnosis [40]. It is therefore difficult to diagnose AS in its early stages relying exclusively on clinical manifestations. This precludes affected individuals from obtaining prompt and targeted treatment. The implementation of genetic testing in clinical practice makes it possible to increase the number of correctly diagnosed patients among those with ambiguous phenotypes caused by rare genetic variants. The development of high-throughput sequencing technologies has enabled clinicians to obtain fast and accurate genetic diagnoses and ensures early multidisciplinary management and treatment of the emerging symptoms.

In this case report of a child with AS by next generation sequencing of a panel of genes linked to eye-related pathologies, the patient was very young (22 months) and did not show any very specific clinical phenotypes, except central loss of visual acuity and accentuated photophobia. We investigated a list of known gene variants associated with degenerative eye diseases. The list can be optimized to include new gene variants or adapted to different groups of disorders by the same approach. Genetic screening of the patient identified two compound heterozygous frameshift variants in *ALMS1*. This established the diagnosis of AS, coherently with our preliminary observation of a slight elevation in body mass index. The first variant p.(Thr399Lysfs*11) was detected in exon 5, the second (p.Glu3771Trpfs*18) in exon 16. Both cause frameshifts that introduce a premature termination codon, like the majority of *ALMS1* variants reported so far [29, 30]. ALMS1 is a long protein of 4167 amino acids. The deletion in exon 5 causes a frameshift at residue 399 and produces a premature stop codon at residue 411, which presumably triggers nonsense-mediated mRNA decay [41]. The variant was recently reported in two brothers with an atypical form of AS [42]. The three base-pair deletion in exon 16 is a known AS variant [29] that causes frameshift at residue 3771 and premature termination at residue 3789, suggesting production of an aberrant protein product lacking the C-terminal domain, which could possibly impair centrosomal localization of ALMS1, as previously reported for other variants [19]. However, in the absence of an analysis of the protein product we cannot exclude that this variant, too, leads to mRNA decay.

Being a pleiotropic disease, treatment of the manifestations of AS requires multidisciplinary management. Useful guidelines for disease management, listing the necessary evaluations after initial diagnosis and therapeutic measures, can be found in GeneReviews® [43]. In brief, since cone-rod dystrophy evolves to complete blindness, early education in orientation and mobility and in the use of Braille is essential to master independent living skills. Hearing loss can be treated with digital hearing aids and cochlear implants. Skeletal complications, often scoliosis, require adequate physiotherapy.

Besides other manifestations, AS results in obesity, severe insulin resistance and dyslipidemia. Through appropriate lifestyle modifications, such as a healthy low-calorie, low fat diet and plenty of physical activity, resulting in weight reduction, it is possible to improve glucose control and serum lipids, lowering the risk of type 2 diabetes, metabolic syndrome and also cardiovascular disease [44–46]. In addition to non-pharmacological management, pharmacological treatments such as statins for managing dyslipidemia [47], ACE-inhibitors in patients with hypertension or left ventricle dysfunction [48] and metformin therapy may be considered for the prevention of type 2 diabetes [49].

Developmental disability needs to be assessed on a case-by-case basis and often requires specialist support from a developmental pediatrician or a pediatric psychiatrist. Good clinical follow-up and full family support can maximize the possibilities of integration in society and educational and employment opportunities.

In conclusion, diagnosis of a very young patient with AS through next generation sequencing offers an example of this method as an exceptionally valuable screening tool for the correct diagnosis patients, especially children, when clinical data is not sufficient for a clear diagnosis. Although whole exome sequencing has proved very useful for accurate early diagnosis of AS [50], the optimization of screening panels based on patient manifestations and the literature may reduce the need for massive screening.

Early genetic diagnosis based on a sensitive and relatively low cost approach allows clinicians to determine the best available therapeutic options without delay, which can be crucial in invalidating rare genetic disorders like AS.

## Supplementary information

**Additional file 1.**

**Additional file 2.**

**Additional file 3.**

## Data Availability

The datasets generated and/or analysed during the current study are available in ClinVar Database (https://www.ncbi.nlm.nih.gov/), c.1196_1202del; p.(Thr399Lysfs*11); ClinVar accession SCV001426409 and c.11310_11313del; p.(Glu3771Trpfs*18); ClinVar accession SCV001426410. Sequence analyses were performed using NCBI Reference Sequence database, GenBank: NM_015120.4. NGS and Sanger sequencing data of the proband and their relatives can be found as supplementary files.

## Abbreviations

AS: Alström syndrome
BBS: Bardet-Biedl syndrome
Ct: Cholesterol
