# Supplementary material for: A very early diagnosis of Alstrӧm syndrome by next generation sequencing
Source: BMC Med Genet. 2020 Sep 1;21:173. doi: 10.1186/s12881-020-01110-1 (PMC7460749; doi:10.1186/s12881-020-01110-1)

**PCR primers used for Sanger sequencing detected pathogenic *ALMS1* variants.**

*ALMS1* NM_015120.4: c.1196_1202del: p.( Thr399Lysfs*11)

| 81_ALMS1_EX5 | TCTCGACCATCGGAAGTTAGTGAA | TGATTACACACGTTTCTGGGTGGT |
| --- | --- | --- |

*ALMS1* NM_015120.4: c.11310_11313del: p.( Glu3771Trpfs*18)

| 123.V1_ALMS1_ex16 | TACCCGTTCTGTCTTCAGGTCAGC | AGCCATGTACCTGGATGTGTCTCC |
| --- | --- | --- |

Sanger Sequencing data

**INDEX PATIENT**

*ALMS1* NM_015120.4: c.1196_1202del: p.( Thr399Lysfs*11) - Heterozygous

TGCTGCTCGTTCATATGGGCAGTATTGGA**CACAGG**AAGATTCATCTAAGCAGGCA


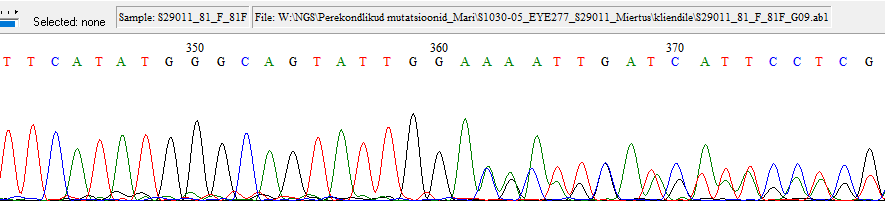


*ALMS1* NM_015120.4: c.11310_11313del: p.( Glu3771Trpfs*18) - Heterozygous

ATCAGATATATTGACCCAAACAGATAG**AGAG**GTGGCTCTGCACGAAAGGAGT


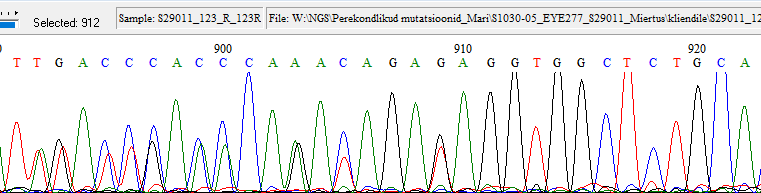


**PROBAND’S MOTHER**

*ALMS1* NM_015120.4: c.1196_1202del: p.( Thr399Lysfs*11) - Heterozygous

TGCTGCTCGTTCATATGGGCAGTATTGGA**CACAGG**AAGATTCATCTAAGCAGGCA


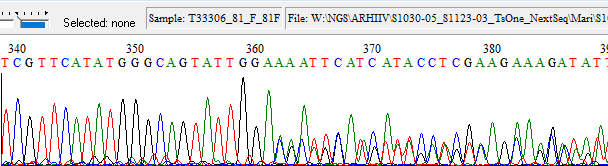


*ALMS1* NM_015120.4: c.11310_11313del: p.( Glu3771Trpfs*18) – wild type

ATCAGATATATTGACCCAAACAGATAG**AGAG**GTGGCTCTGCACGAAAGGAGT


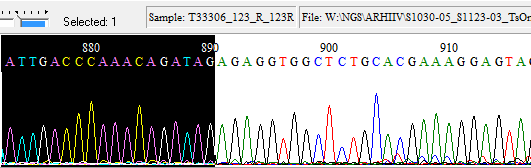


**PROBAND’S FATHER**

*ALMS1* NM_015120.4: c.1196_1202del: p.( Thr399Lysfs*11) – wild type

TGCTGCTCGTTCATATGGGCAGTATTGGA**CACAGG**AAGATTCATCTAAGCAGGCA


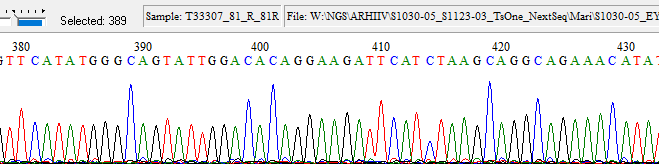


*ALMS1* NM_015120.4: c.11310_11313del: p.( Glu3771Trpfs*18) - Heterozygous

ATCAGATATATTGACCCAAACAGATAG**AGAG**GTGGCTCTGCACGAAAGGAGT


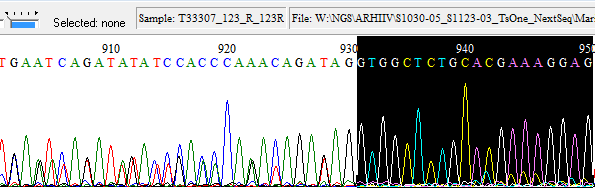


**PROBAND’S SISTER**

*ALMS1* NM_015120.4: c.1196_1202del: p.( Thr399Lysfs*11) – wild type

TGCTGCTCGTTCATATGGGCAGTATTGGA**CACAGG**AAGATTCATCTAAGCAGGCA


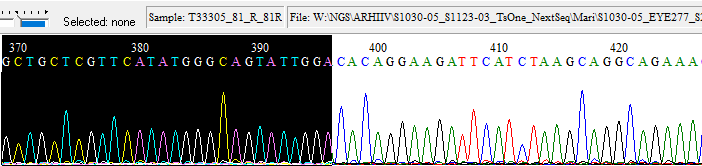


*ALMS1* NM_015120.4: c.11310_11313del: p.( Glu3771Trpfs*18) – wild type

ATCAGATATATTGACCCAAACAGATAG**AGAG**GTGGCTCTGCACGAAAGGAGT


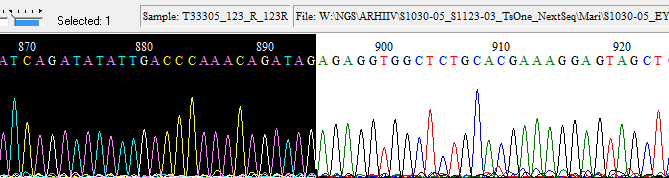

Supplement: Supplementary file 3 — Additional file 3. [file 12881_2020_1110_MOESM3_ESM.docx]
